# The ethics of clinical research in the era of COVID-19

**DOI:** 10.3389/fpubh.2024.1359654

**Published:** 2024-03-06

**Authors:** Juliana N. Vitti, Robert Vitti, Karen Chu, Scott Mellis

**Affiliations:** ^1^Boston University, Boston, MA, United States; ^2^Regeneron Pharmaceuticals Inc., Tarrytown, NY, United States

**Keywords:** clinical trial conduct, COVID-19 therapeutics, COVID-19 vaccines, biospecimens, ethics – clinical

## Abstract

There is an urgent need for increased understanding of COVID-19 and strategies for its prevention, treatment, and mitigation. All participants in the research enterprise, including institutional review boards, have an ethical duty to protect participants and ensure that the benefits gained from such research do not conflict with the core principles that guided researchers prior to the pandemic. In this review, we discuss the ethical issues surrounding initiation and conduct of clinical trials, focusing on novel COVID-19 therapeutic, vaccine, or biospecimen research, using the principles of autonomy, beneficence, and justice. We discuss strategies to manage the practical challenges associated with the conduct of clinical trials, with an emphasis on maintaining the rights and welfare of research participants.

## Background

Clinical trials remain an indispensable part of the healthcare system for the evaluation and development of future therapies and are essential for the approval of new therapies by regulatory authorities for use by the general public. The challenges of the COVID-19 pandemic brought unprecedented risks and raised questions about how to maintain the ethical integrity and fair conduct of clinical trials (1).

The COVID-19 outbreak was officially declared a pandemic by the World Health Organization on March 11, 2020 (2). Globally, as of December 2023, over 772 million cases had been identified and approximately seven million deaths attributed to the infection (3). Widespread full or partial societal lockdowns across much of North America, Europe, and Asia led to far-reaching disruptions in international travel, commerce, and supply chains. As a result, many clinical trials were either suspended or delayed (4), as research shifted in focus toward finding a treatment or vaccine for COVID-19 amid widespread healthcare staff shortages and resource reallocation in both clinical and non-clinical functions. The US National Institutes for Health (NIH) clinical trial registry lists over 200 potential COVID-19 treatment or vaccine trials that had been rapidly initiated by April 2020 (ClinicalTrials.gov; US and ex-US trials can be registered), with thousands either completed or ongoing since then. Against the backdrop of COVID-19 restrictions, the routine ethical conduct of some accelerated trials was potentially challenged by factors such as inadequate numbers of trained research personnel, and limited in-person third-party inspection of trial sites (5). Moreover, many clinical trials, irrespective of whether ongoing or new, and for treatments related or unrelated to COVID-19 alike, faced constraints that necessitated a reimagining of clinical trial conduct.

New guidance on the management of clinical trials has been released and is continuously updated by the European Medicines Agency (6), US Food and Drug Administration (FDA) (7), and several national health authorities (8–10). Following widespread availability of effective vaccines, many of the trials postponed or delayed during the pandemic have now restarted. However, certain patient populations, such as older adult patients or those with particular diseases or who are undergoing treatments that lead to immunosuppression, may still be at risk for developing severe COVID-19 (11–14). The initiation and ongoing conduct of clinical trials should be tailored to the unique risks and burdens participants may encounter for each clinical trial, based on the ethical principles that generally apply to clinical research in humans. This review discusses the challenges facing researchers and sponsors conducting clinical trials in the era of COVID-19 and ways to overcome these that maintain the ethical and scientific integrity of clinical research.

## Clinical research and the need for an ethical framework

Clinical research aims to help researchers discover and understand better ways to detect, treat, and prevent disease by studying health and illness in human participants and/or patients. Ethical guidelines are required to avoid or minimize risks to the patients and/or volunteers and to preserve the integrity of scientific research (15). Abuses of human participants in research in the past, along with advances in medicine and technology and societal changes, have led to development of various codes of ethics by which clinical research is currently conducted. These include the Nuremberg Code (16), Declaration of Helsinki (17), Belmont Report (18) and, more recently, the Declaration of Taipei (19).

In particular, the Belmont Report outlined three basic ethical principles that have shaped the conduct of clinical trials today. The first is respect for the person, acknowledging individuals as autonomous agents, and protecting those with diminished autonomy. The requirement for informed consent as an essential prerequisite for participation in clinical trials stems from this principle. The second principle, beneficence, stipulates that efforts should be made to secure the well-being of research subjects by maximizing the potential benefits while minimizing possible harms for the participants, in line with the basic medical principle of ‘first, do no harm.’ Third, justice applies to the fair and unbiased selection of study participants, so that all eligible people regardless of wealth, disability, gender, race, or ethnicity should have equal opportunity to participate in clinical trials and have research results that are relevant to them (18).

## Impact of COVID-19 on clinical research

Clinical research is essential for determining the safety and efficacy of potential new treatments. While the safety of human research volunteers is the first priority when considering initiating research, certain new and unknown risks to clinical trial participants were introduced or exacerbated by the pandemic; for instance, between different individuals, an active COVID-19 infection might cause a study treatment (unrelated to the treatment of COVID-19) to have no effect or an altered effect. The study treatment might affect the risk that a participant contracts and/or has a more severe COVID-19 infection (1). Trial implementation must ensure that harm is minimized and benefit is maximized in the event of any of these possibilities. Other issues to consider are outlined in Table 1.

**Table 1:** Challenges for clinical trial research during the COVID-19 pandemic.

Challenges	COVID-19 vaccine trials	COVID-19 therapeutic trials	Other drug trials
Increased COVID-19 transmission risk from travel for research participants and staff, medical monitoring, and surgical procedures	+	+	+
Necessity of quarantines and/or social distancing for study participants, study coordinators, clinical staff, and/or site personnel	+	+	+
Closure of investigative sites, laboratories, and/or outpatient monitoring sites	+	+	+
Implementation of travel restrictions	+	+	+
Staffing/supply shortages due to reallocation of clinical staff/supplies to the pandemic response as well as supply chain disruptions	+	+	+
Unrelated study treatment that affects disease course of COVID-19	+	+	−
COVID-19 infection or vaccination affects response to unrelated study drug or condition under investigation	−	−	+
Identification of new viral variants that may affect efficacy of current approved vaccines/treatments	+	+	−
Obtaining ethically appropriate consent for biospecimens from severely ill patients	+	+	−
Maintenance of rigorous study designs and methodology	+	+	+
Minimizing potential bias	+	+	+
Ensuring fair participant selection when rapid patient enrollment is a priority	+	+	−

These challenges also pose the potential risk that it might not be possible to complete a clinical study in a manner that would properly meet the stated research objectives. This is an important consideration as many trial participants may volunteer to be part of a study for altruistic reasons such as the advancement of science or benefit of the larger population, in addition to potential personal benefit. Therefore, out of respect for the participants, a well-designed study must be able to fulfil its stated research objectives.

The FDA has outlined general considerations to assist sponsors in assuring the safety of trial participants, compliance with good clinical practice, and minimization of risks to trial integrity (7). From our own experience of clinical trials conducted or initiated during the pandemic, risk minimization measures implemented to assure safe continuation of the trial included requiring COVID-19 testing as part of the initial screening, designing the study to reduce the number of visits, or allowing study procedures, such as blood draws or administration of questionnaires, to be completed at additional facilities or even at home. Study sponsors also quickly modified operational procedures to include remote oversight, patient monitoring, and data collection, to minimize the need for in-person visits of non-essential personnel to healthcare facilities. Some institutions introduced mandatory screening of all patients for COVID-19 exposure and symptoms of illness, including research participants, which ensured screening of patients in clinical trials without needing individual researchers to obtain institutional review board (IRB) approval for COVID-19 screening in each study (20). Similar recommendations for minimizing risks to trial participants have been made by other national health authorities, such as the UK Medicines and Healthcare Products Regulatory Agency (MHRA) (8).

### Application of ethical principles to clinical research during COVID-19

#### Respect for persons: obtaining informed consent

##### Applications in vaccine trials

The Belmont Report established that research subjects must freely consent to participate, and need to know and understand the nature of the research, what will happen to them, and the potential risks and benefits (18). Study-specific informed consent, i.e., consent obtained from all participants before each study is undertaken and that only covers the specific study in question, has been the standard in research involving human subjects (21). This approach was used in the major vaccine trials, in which participants were provided with a presentation of key information regarding the clinical trial, both verbally and in written form. Participants were asked to read and review the consent form, and signatures were obtained before any study procedures were performed (22–24).

##### Applications in studies evaluating COVID-19 therapeutics

In studies for therapies for COVID-19, consent procedures varied, depending on the patient population. Some trials, such as the TOGETHER and REGEN-COV antibody trials, used a traditional signed consent form approach (25, 26). However, for patients who had been hospitalized, obtaining consent at the time of study initiation was sometimes challenging. Prior to the pandemic, the FDA had issued guidelines for the exception of informed consent in cases of emergency research. Emergency research was defined as investigations involving human subjects who have a life-threatening medical condition that necessitates urgent intervention and who, because of their condition, cannot provide informed consent (27). With this precedent in mind, studies evaluating outcomes in hospitalized patients needed to develop more flexible protocols to deal with cases in which the clinical course of patients may rapidly change, or where friends or family who may be designated as healthcare proxies, are not allowed to enter the facility.

The RECOVERY protocol was designed to evaluate the efficacy of administration of dexamethasone plus usual care to hospitalized patients with COVID-19, compared with usual care alone, while minimizing the burden on front-line hospital staff. The adaptive protocol specified that if the patient cannot give consent due to the severity of their illness, then consent may be obtained from a legal representative or independent clinician. Consent from the patient would then be obtained if they recovered (28).

##### Applications to biospecimen research

Biospecimen research has become another strategy to better understand COVID-19 disease, improve testing, aid in genomic identification of new variants, and inform clinical trials assessing treatment and prevention strategies, including ongoing vaccine development. While study-specific informed consent may be appropriate for samples collected during a study with prespecified objectives and retention time, biospecimen research may involve uses that are unknown at the time of initial consent. A policy of broad or general consent has been proposed whereby a participant may be asked to provide consent that permits the collection, storage, and secondary use of biospecimens for future research purposes (29, 30). The decreased level of protection provided by this model has been criticized, primarily because the difficulty of predicting future research options can hide ethical challenges from participants, ultimately eroding trust towards researchers (31). However, a systematic literature review of attitudes regarding biospecimen research, including consent for data storing and sharing, revealed that participants often preferred broad consent over study-specific consent. This was particularly true when broad consent was the only option, samples were de-identified, logistics were communicated, and privacy issues were addressed (32). A revised broad consent model has been proposed, which relies on a strong and continuous ethical review process, including specific mandates for proposed research that considers whether the research is within scope and allows for continuous provision of information to participants (33).

In response to the pandemic, New York University Langone Health (NYULH) amended an existing IRB–approved universal consent protocol to allow for a temporary waiver of consent for the prospective collection of research biospecimens and corresponding clinical data from patients presenting with COVID-19 for research purposes at any NYULH facility (34). The protocol specified that, for living patients, waivered consent remained in effect until their clinical condition stabilized and until obtaining consent did not create additional risk for the patient and/or research support staff at the patient’s next clinical visit at NYULH. If a patient denied consent, banked specimens were to be destroyed and recorded data removed from the clinical database. In the event the patient died before they could provide consent, banking of de-identified leftover specimens and clinical data was permitted (34).

The advantages of this or similar approaches include enhancing patient protections and obtaining population-level data, minimizing interruptions in the delivery of care, lowering potential exposure risk by reducing transportation of biohazardous materials, maximizing the number of specimens collected, allowing adherence to biosafety guidelines, and providing the possibility for generation of a statistically powered, standardized cohort characterization by compiling results in one biobank (34). While this approach ensures broad patient representation in sample collection, it had the potential, during the pandemic, to create additional ethical issues due to the rapidly developing nature of the virus and uncertainty surrounding resumption of in-person visits. Ensuring appropriate follow-up and ongoing informed consent was also challenging due to altered social circumstances during the pandemic, such as unemployment and changes in residence, which could make contacting individuals difficult. An additional consideration for future trials is that, if a patient does not provide consent under this framework but the samples have already been sequenced and uploaded into a public repository, removal may not be possible.

#### Beneficence: maximizing possible benefits of COVID-19 research while minimizing harms

##### Applications in vaccine trials

Study sponsors have an ethical responsibility to minimize risk. Those conducting the three major placebo-controlled vaccine trials discussed here sought to fulfil this requirement through various methods, such as using a staggered enrollment strategy, stratifying patients by age and/or health risk prior to randomization, implementation of an independent data monitoring committee to oversee continuous efficacy and safety assessments, and active adverse event monitoring and COVID-19 symptom surveillance (22–24). Additionally, in recognition of the possibility that a vaccine might become available under an Emergency Use Authorization before completion of the study, Pfizer-BioNTech, Moderna, and Janssen stipulated that placebo recipients who chose not to remain in the ongoing study and were eligible for an authorized/licensed vaccine would need to be unblinded (24, 35, 36).

##### Applications to biospecimen research

There are a number of risks associated with biospecimen research, including violation of patient privacy, breach of confidentiality, and exploitation of protected and vulnerable groups. Participation in biospecimen research does not offer individuals the prospect of direct benefit, but rather the social benefits of advancements in treatment within a relatively short period and the possibility of timely influence on mitigating disease and/or altering its course. IRB oversight of COVID-19 biospecimen research requires adherence to the maximum level of privacy and confidentiality for participants while minimizing risks and ensuring that a study has equitable selection of subjects, appropriate consent processes, effective and clear cultural and multilingual communications, community engagement, and mechanisms in place to identify and address stigmatization. As a minimum, the IRB should conduct a review of research as appropriate to the degree of risk at least once a year (37); however, given the evolving ethical landscape, more frequent reviews may be necessary.

#### Justice: fair and unbiased selection of clinical research subjects

##### Applications in studies evaluating COVID-19 therapeutics

The Belmont Report stipulates that the benefits of publicly funded research ought to be fairly available to all (18); however, there is no specific guidance on how to facilitate this. Applying the principle of justice as conceived in the Belmont Report, IRBs have traditionally focused on equitable recruitment and inclusion. Once the rapid spread of COVID-19 began overwhelming hospital systems, there was an urgent need to find treatments for COVID-19. As healthcare professionals used existing therapies to try and improve outcomes, patients were rapidly recruited to assess the overall efficacy of these therapies and provide treatment guidelines. However, recruitment most often was not representative of the overall population. In clinical trials evaluating the efficacy of REGN-COV compared with usual care or placebo, respectively, over 80% of patients identified as White versus Black, Asian, or another minority group (26), but people who identify as White make up a smaller proportion (62%) of the current US population (38). People of Native American, Black, and Hispanic/Latino descent are 3.5, 2.8, and 2.8 times at greater risk of hospitalization, respectively, and at 2.4, 2.0, and 2.3 times greater risk, respectively, of death from COVID-19 compared with people who identify as White (39), and therefore arguably should be represented to a greater extent in trials for COVID-19 therapies.

##### Applications in vaccine trials

A trend towards decreased representation of minorities was also seen in the phase 3 trials of the two-dose Moderna and Pfizer-BioNTech vaccines, with over 79% of participants identifying as White (22, 23). By contrast, the phase 3 trial evaluating the safety and efficacy of the Janssen single-dose vaccine had a more racially diverse population, with 59%, 45%, and 19% of participants identifying as White, Hispanic, and Black, respectively (24). The increased enrollment of minority groups could be due to a number of factors, including that the trial was conducted later in the pandemic (enrollment began in September 2020). Other factors that could have played a role include the increasing magnitude of the pandemic as new variants fueled additional surges in various regions, the reporting by US states of racial and ethnic data along with data on cases and deaths, location of the trial (previous trials were conducted in centers primarily in the US, whereas this trial incorporated additional centers in various countries throughout South America), and more convenient administration (single vs. two doses).

In addition to the underrepresentation of minority groups in these trials, vulnerable groups such as pregnant or nursing women were excluded. Although this is often the case in large-scale clinical trials of a new therapeutic or vaccine following risk–benefit assessments, other new pathogens (such as the Zika virus) have demonstrated an increased risk of complications in pregnant women and birth defects in their offspring (40, 41). Accumulating observational data during the course of the COVID-19 pandemic has shown high rates of hospitalization, pre-term delivery, and cesarean section (42). This underscores the need for clinical studies of vaccines and treatments to include pregnant and nursing women in the study population. In light of the need for prospective clinical trial data in this unique population, recommendations have been developed that provide a roadmap for the ethically responsible, socially just, and respectful inclusion of the interests of pregnant women in the development and deployment of vaccines against emerging pathogens (43); these should be taken into consideration during the design of any future clinical vaccine or therapeutics trials.

## Clinical trial design/conduct

As with all trials, those involving COVID-19 vaccines/therapeutics should have a rigorous design and be adequately powered to generate clinically meaningful data. There is an inherent risk that a poorly controlled trial could yield misleading results and lead to the promotion of therapies that could be ineffective or result in serious harm to the general population. This was seen early in the pandemic with the promotion of hydroxychloroquine as a possible effective treatment for COVID-19, based on a small open-label study suggesting that therapy with hydroxychloroquine and azithromycin decreased viral load (44, 45). This inflated public belief of the drug’s benefits led to increased public demand, with some physicians prescribing it for prophylaxis and/or outpatient treatment of COVID-19, leading to widespread shortages and inability to fill prescriptions for patients with chronic conditions for which it is approved, such as lupus (44). However, the majority of evidence for the use of chloroquine/hydroxychloroquine thus far has been from both *in vitro* and clinical studies of low methodological quality. The latter studies had several issues, including but not limited to small sample sizes, lack of a control arm, confounding bias in observational studies, unadjusted analyses, and/or unclear reporting of methods (46). Additionally, a recent meta-analysis found that treatment with hydroxychloroquine is associated with increased mortality in COVID-19 patients, while treatment with chloroquine presented no benefit (47).

Although randomized controlled trials remain the gold standard for efficacy data, study sponsors may consider alternative designs that allow the study to be adequately controlled while remaining ethical and not posing an undue burden or additional risk on the patients randomized to the control group. These include but are not limited to: conducting a head-to-head trial of a candidate therapy against an existing, approved, equally efficacious therapy (active control); deferring immunization from one group (blinded crossover); and active and passive observational studies of randomized controlled trial participants and population-based cohorts. The use of novel trial designs may also be considered, such as the stepped-wedge design, which involves random and sequential crossover of clusters from control to intervention until all clusters are exposed to the study drug (48), or the platform design, which evaluates several interventions against a common control group and can be perpetual. Additionally, this design has prespecified adaptation rules to allow dropping of ineffective intervention(s) and flexibility of adding new intervention(s) during the trial (49).

### Secondary vaccine trials

Once Emergency Use Authorizations and/or approvals were received for the Comirnaty (Pfizer-BioNTech) (50), Moderna (51), and Janssen (52) vaccines, the ethical burden shifted to other sponsors who wished to conduct placebo-controlled trials in light of the availability of efficacious and presumably safe vaccines. These secondary vaccine trials presented unique characteristics that require bioethical considerations. There are arguments that placebo-controlled trials conducted during a pandemic are ethically impermissible if an efficacious vaccine is available (53), as such trials would involve withholding the vaccine from people in the control group who have a moral right to immunization. Maintenance of a placebo or control group might also limit the effectiveness of wider efforts to contain the pandemic. There are still instances, however, where placebo-controlled randomized controlled trials with vaccine candidates might be ethically acceptable, such as those performed with populations not yet eligible for the authorized vaccines (e.g., children, pregnant women), those in which vaccine efficacy is a secondary endpoint, or those that evaluate a new indication, such as booster or multiple doses in people who have already been vaccinated (53). Beyond efficacy, there may be additional reasons why a candidate vaccine may be more valuable, such as ease of administration, more convenient storage temperatures, or usefulness as a backup should the supply of existing vaccines be threatened. Secondary vaccine trials, are therefore, necessary, and resolution of the associated ethical issues is important. Additionally, sponsors of trials for new investigational COVID-19 treatments need to consider the inclusion of both previously vaccinated and unvaccinated participants as part of the study population, as well as the potential for differential efficacy and safety across these groups. Although the development timelines for COVID-19 vaccines and treatments were exceptionally rapid, consideration of these factors may slow the development of future treatments.

## Conclusion

The COVID-19 pandemic presented unique challenges and raised ethical questions concerning the conduct of clinical research, yet it remains vital to ensure new therapies are being evaluated both for patients with COVID-19 and without COVID-19. During the course of this ongoing pandemic, ethical issues emerged for clinical trials of all types and in various therapy areas, as well as vaccines and treatments specifically to combat COVID-19. The need for continuous evaluation of whether the principles of respect for persons (informed consent), beneficence, and justice were being adhered to during a time of unprecedented global disruption to healthcare and society was, and still is, critical. Additionally, adaptation was needed to ensure ethically appropriate consent for collection of biospecimens from patients in real-world clinical settings to allow for genomic characterization of COVID-19, with possible translational application beyond the current crisis. Given the rapid development and subsequent approval of vaccines, sponsors and IRBs overseeing additional new COVID-19 vaccine and therapeutics trials will have to consider whether the scope of potential benefits gained outweigh the reasons for not initiating the trial against the backdrop of dominant local/worldwide strains, regional availability, efficacy of approved vaccines, number of cases, and strains on healthcare resourcing.

## Author contributions

JV: Writing – original draft, Writing – review & editing. RV: Writing – original draft, Writing – review & editing. KC: Writing – original draft, Writing – review & editing. SM: Writing – original draft, Writing – review & editing.
